# The Crystal Structure of the Domain of Unknown Function 1480 (DUF1480) From *Klebsiella pneumoniae*

**DOI:** 10.1002/prot.26752

**Published:** 2024-09-26

**Authors:** Dhruvin H. Patel, Nobuhiko Watanabe, Alexei Savchenko, Cameron Semper

**Affiliations:** ^1^ Department of Microbiology, Immunology and Infectious Diseases University of Calgary Calgary Alberta Canada; ^2^ Center for Structural Biology of Infectious Diseases (CSBID) Calgary Canada

**Keywords:** antibiotic response, DUF1480, *Klebsiella pneumoniae*, X‐ray crystallography

## Abstract

Domains of unknown function (DUFs) continue to comprise a significant portion of bacterial proteomes, with more than 20% of bacterial proteins remaining annotated as DUFs. The characterization of their molecular structure can provide valuable insight that is not captured by the primary sequence analysis, thus providing a segue into the identification of the molecular function of DUF representatives. Here, we present the crystal structure of KPN_02352 from *Klebsiella pneumoniae* subsp. *pneumoniae*, a DUF1480 domain‐containing protein, which was determined to be 1.75 Å resolution. Representatives of the DUF1480 family are found broadly across *Enterobacterales* and have been previously shown to contribute to the antibiotic response. Our structural analysis suggests that DUF1480 is comprised of a six‐stranded split barrel fold featuring a small alpha helix that is positioned to cap one end of the split barrel. DUF1480 was found to be monomeric in solution, and harbors structural similarity to response regulators. The crystal structure of DUF1480 is the first experimental insight into the molecular structure of this conserved protein family, revealing several conserved features that may be functionally relevant.

## 1 Introduction

The rise in available genomic and proteomic data has led to the identification of a vast array of conserved protein domains and proteins for which the biological function is unknown. These domains of unknown function (DUFs) can be found across all domains of life, and it is estimated that approximately 20% of all proteins are categorized as DUFs [1]. DUFs are particularly common in bacterial species where there are currently more than 2500 known bacterial DUFs. A recent analysis of 16 model bacterial species found that 355 essential gene products contained a DUF or were solely comprised of a DUF, suggesting these uncharacterized proteins play important roles in critical biological processes [1]. Moreover, a number of studies have shown that DUFs or DUF‐containing proteins can play important roles in virulence [2], toxin‐antitoxin systems [3], pathogenesis [4], and antimicrobial resistance [5]. This highlights the need for continued investigation into DUFs including strategies that can help uncover insights into their biological functions, such as structural and biochemical characterization.

DUF1480 is represented by 78–80 amino acid proteins found in 831 species across the bacterial kingdom, including pathogenic species in *Enterobacterales*. While the members of this family have seldom been investigated outside high‐throughput screening studies, the available data hints at a diverse and important biological function of this protein. In single‐gene knockout experiments in the KEIO collection, *E.coli K‐12* harboring a YebV/DUF1480 deletion was shown to display increased sensitivity to metronidazole, ampicillin, triclosan, and cefoxitin [5]. DUF1480 was shown to be tightly controlled by RpoS/σ^S^, a master regulator of general stress response, in the human pathogen *Salmonella enterica* serovar Typhimurium [6]. The DUF1480‐containing protein YebV was found enriched in the secretome of polymyxin B‐resistant *E.coli*, further implicating this family of proteins in antimicrobial response [7]. High‐throughput protein–protein interaction studies in *E*. *coli* identified a number of transcriptional regulators, including FIS, kdgR, purR, and yciT, as interactors of YebV/DUF1480 [8]. Taken together, these data point to a role for DUF1480 in cell stress response; however, further investigation is required to elucidate the role of these conserved proteins.

Here, we present the high‐resolution crystal structure of KPN_02352, a DUF1480 domain‐containing protein from the human pathogen *Klebsiella pneumoniae* subsp. *pneumoniae*. Our structure reveals that KPN_02352 consists of a fold primarily comprised of *β*‐strands that form a split barrel arrangement. A broader analysis of the DUF1480 family showed that the protein is highly conserved, with even the most distant orthologs sharing ~87% sequence identity. Using our crystal structure, we identified structural similarities between DUF1480 and other cellular response regulators, pointing to a potential functional role for this family of proteins.

## 2 Materials and Methods

### 2.1 Cloning, Expression, and Purification of DUF1480

The KPN_02352 ORF was PCR amplified from *K. pneumoniae* subsp. *pneumoniae* genomic DNA and cloned into the pMCSG53 [9] vector via T4 ligation‐independent cloning. The plasmid was transformed into BL21 Gold (Agilent) for protein expression. Protein purification proceeded as described in a previous study [10]. Briefly, the cells were grown at 37°C and 175 rpm until the OD_600_ reached 0.8, after which the cells were induced by the addition of IPTG to a final concentration of 0.5 mM. Cells were incubated at 20°C for 16 h, then harvested via centrifugation and resuspended in binding buffer [300 mM NaCl, 50 mM HEPES pH 7.5, 5 mM imidazole, 5% glycerol]. Cells were lysed via sonication, clarified via centrifugation, and then incubated with Ni‐NTA resin for 1 h. KPN_02352 was eluted in elution buffer [300 mM NaCl, 50 mM HEPES pH 7.5, 250 mM imidazole, 5% glycerol], and then incubated with tobacco‐etch virus (TEV) protease to cleave the 6His tag while being dialyzed to remove imidazole. The protein was passed over a second Ni‐NTA column and then dialyzed into a crystallization buffer [300 mM NaCl, 10 mM HEPES pH 7.5]. For SelMet‐derivatized protein, the protein was expressed in M9 selenomethionine medium for *E. coli* (Shanghai Medicilon) without deviation from the manufacturer's protocol. Purification of SelMet‐DUF1480 proceeded as described above.

### 2.2 Crystallization

Crystals of the native and Selenomethionine‐derivatized protein were grown at 298 K in 25% PEG 3350/0.1 M citric acid pH 3.5 via the vapor diffusion sitting‐drop method. Prior to data collection, crystals were soaked in 25% PEG 3350/0.1 M citric acid pH 3.5/30% glycerol and then frozen in liquid nitrogen.

### 2.3 Data Collection, Structure Determination, and Refinement

The data set for the Selenomethionine‐derivatized crystals of KPN_02352 was collected at 100 K on the 21‐1D‐F beamline at the Advanced Photon Source at Argonne National Laboratory. Diffraction data was processed using HKL‐3000 [11] and the structure was solved using single‐wavelength anomalous diffraction (SAD) using PHENIX.solve [12]. The model was built using PHENIX.autobuild [12] and manually refined using Coot [13]. PyMOL was used to generate the figures. Structure factors and coordinates for DUF1480 were deposited to the Protein Data Bank under the identifier 8VVA.

### 2.4 Structure Analysis

Searches for structural homologs of KPN_02352 were performed using Foldseek [14] and DALI [15] server. Electrostatic surface potential was calculated using APBS [16] in PyMol. Consurf [17] was used to map amino acid conservation onto the structure, which was then visualized using PyMol.

## 3 Results

In an effort to structurally characterize DUF1480 representatives, we purified the DUF1480 domain‐containing proteins YebV from *E. coli* and KPN_02352 from *Klebsiella pneumoniae*. The native *K. pneumoniae* KPN_02352 protein was crystallized in 25% PEG 3350/0.1 M citric acid pH 3.5. Interestingly, we were unable to produce crystals of the *E. coli* YebV protein despite a high level (87%) of shared amino acid sequence identity between the two DUF1480 orthologs. We used a SelMet‐derivatized protein sample, which crystallized under the same conditions, to determine the structure of KPN_02352 by the SAD approach. The structure was further refined to a resolution of 1.75 Å. The crystallographic statistics are summarized in Table 1.

**TABLE 1:** X‐ray crystallographic statistics.

	KPN_02352 (PDB: 8VVA)
Data collection	
Space group	*P*4_1_2_1_2
Cell dimensions	
*a*, *b*, *c* (Å)	78.591, 78.591, 62.492
*α*, *β*, *γ* (°)	90.0, 90.0, 90.0
Wavelength	0.9792
Resolution (Å)	41.53–1.75
	(1.78–1.75)
*R*_meas_ ^1^	0.108 (2.173)
*R*_pim_ ^2^	0.036 (0.667)
*I*/s*I*	14.1 (1.7)
Completeness (%)	99.7 (96.7)
Redundancy	11.1 (10.7)
CC1/2	0.971 (0.320)
Refinement	
Resolution (Å)	33.27–1.75
No. reflections	20 271
*R*_work_/*R*_free_ ^3^	0.2049/0.2391
No. of atoms	
Protein	1221
Water	65
Average B‐factors (Å^2^)	31.88
Protein	31.58
Water	37.51
R.m.s deviations	
Bond lengths (Å)	0.0087
Bond angles (°)	1.18
Ramachandran (%)	
Favored	99.33
Allowed	0.67
Disallowed	0

*Note*: Values in brackets refer to highest resolution shells.

^1^ *R*_meas_ = ∑_hkl_√ (*n*/*n* − 1) ∑^*n*^_j = 1_|*I*_hkl.j_ − ⟨*I*_hkl_⟩|/∑_hkl_∑_j_ *I*_hk,j_.

^2^ *R*_pim_ = ∑_hkl_√ (1/*n* − 1) ∑^*n*^_j = 1_|*I*_hkl.j_ − ⟨*I*_hkl_⟩|/∑_hkl_∑_j_ *I*_hk,j_.

^3^ *R* = ∑|*F*_*p*_^obs^ − *F*_*p*_^calc^|/∑*F*_*p*_^obs^, where *F*_*p*_^obs^ and *F*_*p*_^calc^ are the observed and calculated structure factor amplitudes, respectively.

**FIGURE 1:**
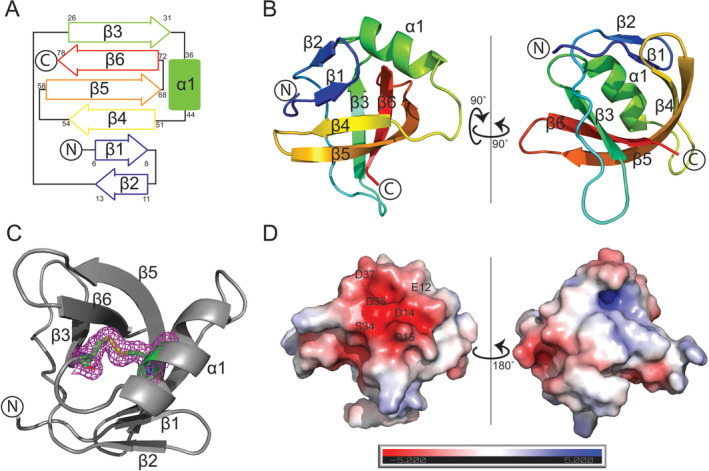
The structure of KPN_02352/DUF1480. (A) Topological arrangement of KPN_02352/DUF1480. (B) Cartoon representation of KPN_02352 colored from N‐terminus (blue) to C‐terminus (red). (C) Disulfide bond found in KPN_02352. The protein is shown in the cartoon representation, while C32 and C39 are shown in the stick representation. The F_o_‐F_c_ map corresponding to the electron density around the disulfide bond is shown in magenta. (D) Electrostatic surface potential of KPN_02352.

The overall structure of KPN_02352 features an antiparallel *β* sheet consisting of six strands with a small *α*‐helix formed on the loop connecting the *β*‐strands three and four (Figure 1A). The *β*‐strands are arranged into a split barrel arrangement allowing for the formation of a hydrophobic core. The split barrel initiates in two short *β*‐strands (*β*1 and *β*2) in an antiparallel arrangement separated by a short hairpin (Figure 1B). A long coil spans the split portion of the barrel leading into *β*3 which establishes the other “wall” of the split barrel. Just prior to *β*3, one of two loops that protrude outward from the structure is present. A short coil after *β*3 leads to *α*1, the lone alpha helix found in the KPN_02352 structure. The *α*‐helix is found adjacent to one end of the split barrel motif and is anchored in place by a disulfide bridge formed between C32 and C39 residues (Figure 1C). After *α*1, a coil leads to the *β*4‐strand, which interacts with *β*1 and *β*5 to establish the contiguous “back” portion of the split barrel. A short hairpin turn separates *β*4 from *β*5, the longest strand in the structure, and another turn leads to *β*6 which is sandwiched between *β*4 and *β*5. The loop between *β*5 and *β*6 is the other loop found protruding outward from the core structure of the protein. Of note is the rotated arrangement of *β*5 which allows for it to form hydrogen bonds with both *β*3 and *β*6 and serve as the “linchpin” of the split barrel arrangement. Analysis of the electrostatic potential of the surface of the KPN_02352 structure showed it to be a primarily electroneutral molecule. The one exception is a small electronegative patch found along the split portion of the barrel near the alpha helix (Figure 1D). The electronegative patch was comprised of the sidechains of residues E12, D14, D15, S34, D35 and D37 (Figure 1D).

**FIGURE 2:**
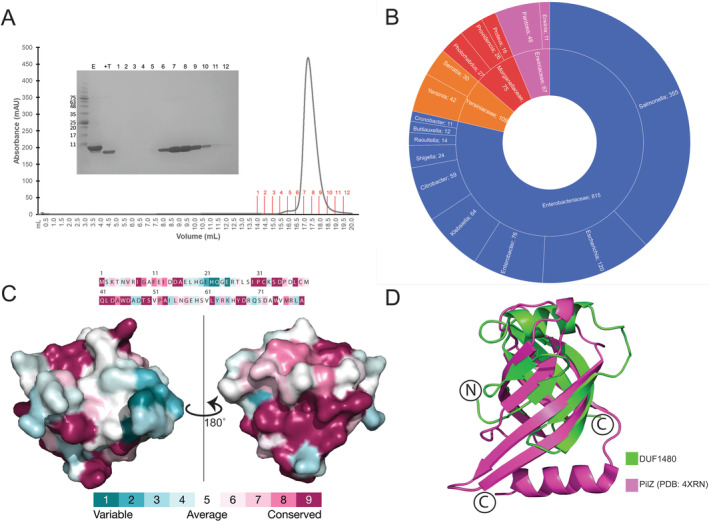
Structure analysis of KPN_02352/DUF1480. (A) Size exclusion chromatogram of KPN_02352. Fractions collected are demarcated in red on the chromatogram, and the SDS‐PAGE inset numbers correspond to these fractions. The result shows KPN_02352 is monomeric in solution. (B) The distribution of DUF1480 in *Enterobacterales*. (C) Consurf analysis of KPN_02352/DUF1480 based on a multiple sequence alignment, showing the distribution of conserved residues throughout the protein. (D) Structural comparison of a pilZ domain from *P. aeruginosa* (PDB: 4XRN) with KPN_02352 (PDB: 8VVA).

KPN_02352 crystallized with two molecules in the asymmetric unit, arranged in a head‐to‐head manner. To investigate the potential biological significance of this arrangement, we analyzed the KPN_02352 oligomerization state in solution using size exclusion chromatography (SEC). Our results showed that KPN_02352 is monomeric in solution, producing a monodisperse peak corresponding to the expected monomeric size of 8.96 kDa (Figure 2A). Of note, both monomers found in the asymmetric unit contained the C32–C39 disulfide bridge. While this feature is expected to represent an artifact of crystallization, given the protein is expected to be localized to the reductive environment of the bacterial cell's cytoplasm, it locks the *α*1‐helix in place where it occludes one end of the split barrel.

As mentioned above, the representatives of DUF1480 are broadly distributed among *Enterobacterales* species and are found in a number of human pathogens including *K. pneumoniae*, *S. enterica*, *Yersinia pestis*, *Shigella sp*., and *Proteus mirabilis* (Figure 2B). Analysis of primary sequence across DUF1480 representatives revealed significant conservation; a pairwise alignment of two of the most phylogenetically diverse orthologs found within this protein family (*K. pneumoniae* and *Mezorhizobium sp*.) showed that these two proteins still share 87.3% amino acid sequence identity. To further analyze the conservation within this protein family we mapped the positional conservation onto our KPN_02352 crystal structure using Consurf [17]. The results of this analysis show high levels of conservation throughout the protein (Figure 2C). Many of the amino acids localized to key secondary structural elements are highly conserved or invariant, including 7/8 of the residues forming the *α*1‐helix. Both the cysteine residues involved in disulfide bond formation (C32 and C39) were highly conserved, but not invariant, suggesting that the disulfide bridge used to stabilize the position of *α*1 relative to the split barrel may not be essential for the maturation of the protein into its tertiary structure. Our analysis identified a small tract of variability localized to the loop region immediately prior to *β*3. This loop protrudes outward from the structure along the same face as one of the “walls” of the barrel.

To further examine KPN_02352 and DUF1480 more broadly we performed searches for structural homologs to the specific fold found in our crystal structure. The majority of the hits identified were uncharacterized proteins harboring a split barrel domain. One functionally annotated family of proteins that were identified as structurally similar to DUF1480 was that of the pilZ domain. Direct comparison between KPN_02352 and a pilZ domain from *Pseudomonas aeruginosa* (PDB: 4XRN) showed that despite the proteins sharing low (~21%) amino acid sequence identity, they share a similar split barrel structure that aligned with an RMSD value of 2.06 Å across 78 C*α* residues (Figure 2D). Proteins that contain pilZ domains have been shown to be response regulators, binding to c‐di‐GMP and then commonly influencing bacterial motility [18, 19]. The pilZ domain typically spans ~110 residues allowing for additional secondary structure elements lacking in DUF1480. Notable features present in KPN_02352, such as the disulfide bond and the localized patch of electronegativity on the surface, are not found in the pilZ crystal structure. Most importantly, the canonical c‐di‐GMP binding motif appears to be lacking in KPN_02352 and DUF1480 in general, indicating the two protein families very likely diverge functionally, at least with respect to metabolite binding.

## 4 Discussion and Conclusions

Despite increasingly sophisticated tools for in silico analysis of genomic data, a significant proportion of protein families remain uncharacterized and annotated as DUFs. Here, we report the high‐resolution crystal structure of *K. pneumoniae* KPN_02352/DUF1480, a conserved DUF found widely within *Enterobacterales*. Our structure revealed that KPN_02352 is comprised of a compact split barrel domain capped on one end by a small *α*‐helix. The barrel featured a hydrophobic core with a sole *α*‐helix anchored in place via a disulfide bond formed between two cysteine residues (C32 and C39). A broader analysis of annotated DUF1480 sequences suggested that the members of this family retain a high level of primary sequence conservation, suggesting that the overall fold is critical for the function of this protein.

The crystal structure of DUF1480 did not provide direct hints about the molecular function of this protein. Our analysis did not reveal any obvious candidates for functionally important residues. The existing literature suggested that STM14_2239 (*S. enterica* DUF1480 domain‐containing protein) gene expression is controlled by RpoS/σ^S^ which is involved in stress response, and DUF1480 has been linked to bacterial response to antibiotics and interactions with a number of transcriptional regulators in other species [5, 6, 8]. Based on this information, we hypothesized that DUF1480 may be functioning as a cell stress response regulator. Our search for structural homologs identified the pilZ domain as the closest functionally characterized structural homolog. PilZ domains are split barrel domains that bind c‐di‐GMP, an important second messenger in cells, resulting in changes to bacterial motility. The identification of a structural homolog playing a role as a response regulator supports our hypothesis that DUF1480 may function as a stress response regulator; however, additional experimentation is required to further characterize the specific role of DUF1480.

## Author Contributions

**Dhruvin H. Patel:** investigation, formal analysis. **Nobuhiko Watanabe:** formal analysis. **Alexei Savchenko:** supervision, funding acquisition, writing – review and editing, conceptualization. **Cameron Semper:** conceptualization, investigation, writing – original draft, writing – review and editing, formal analysis, supervision.

## Conflicts of Interest

The authors declare no conflicts of interest.

### Peer Review

The peer review history for this article is available at https://www.webofscience.com/api/gateway/wos/peer‐review/10.1002/prot.26752.

## Data Availability

The structure of KPN_02352/DUF1480 and all associated data have been deposited to the protein databank (www.rcsb.org) and are available under the accession code PDB: 8VVA.

## References

[1] N. F. Goodacre , D. L. Gerloff , and P. Uetz , “Protein Domains of Unknown Function Are Essential in Bacteria,” MBio 5, no. 1 (2013): e00713–e00744. 10.1128/mBio.00744-13 PMC3884060 24381303

[2] G. Condemine and B. Le Derout , “Identification of New Dickeya Dadantii Virulence Factors Secreted by the Type 2 Secretion System,” PLoS One 17, no. 4 (2022): e0265075. 35417462 10.1371/journal.pone.0265075 PMC9007343

[3] T. Kurata , C. K. Saha , J. A. Buttress , et al., “A Hyperpromiscuous Antitoxin Protein Domain for the Neutralization of Diverse Toxin Domains,” Proceedings of the National Academy of Sciences of the United States of America 119, no. 6 (2022): e2102212119. 35121656 10.1073/pnas.2102212119 PMC8832971

[4] B. Lobb , B. J. Tremblay , G. Moreno‐Hagelsieb , and A. C. Doxey , “PathFams: Statistical Detection of Pathogen‐Associated Protein Domains,” BMC Genomics 22, no. 1 (2021): 663. 34521345 10.1186/s12864-021-07982-8 PMC8442362

[5] A. Liu , L. Tran , E. Becket , et al., “Antibiotic Sensitivity Profiles Determined With an Escherichia Coli Gene Knockout Collection: Generating an Antibiotic Bar Code,” Antimicrobial Agents and Chemotherapy 54, no. 4 (2010): 1393–1403. 20065048 10.1128/AAC.00906-09 PMC2849384

[6] M. Lago , V. Monteil , T. Douche , et al., “Proteome Remodelling by the Stress Sigma Factor RpoS/Sigma(S) in Salmonella: Identification of Small Proteins and Evidence for Post‐Transcriptional Regulation,” Scientific Reports 7, no. 1 (2017): 2127. 28522802 10.1038/s41598-017-02362-3 PMC5437024

[7] D. H. Yang , S. Liu , L. Cao , et al., “Quantitative Secretome Analysis of Polymyxin B Resistance in Escherichia Coli,” Biochemical and Biophysical Research Communications 530, no. 1 (2020): 307–313. 32828304 10.1016/j.bbrc.2020.07.010

[8] M. Babu , R. Arnold , C. Bundalovic‐Torma , et al., “Quantitative Genome‐Wide Genetic Interaction Screens Reveal Global Epistatic Relationships of Protein Complexes in Escherichia Coli,” PLoS Genetics 10, no. 2 (2014): e1004120. 24586182 10.1371/journal.pgen.1004120 PMC3930520

[9] W. H. Eschenfeldt , M. Makowska‐Grzyska , L. Stols , M. I. Donnelly , R. Jedrzejczak , and A. Joachimiak , “New LIC Vectors for Production of Proteins From Genes Containing Rare Codons,” Journal of Structural and Functional Genomics 14, no. 4 (2013): 135–144. 24057978 10.1007/s10969-013-9163-9 PMC3933008

[10] C. Semper and A. Savchenko , “Protein Expression and Purification of Bioactive Growth Factors for Use in Cell Culture and Cellular Agriculture,” STAR Protocols 4, no. 3 (2023): 102351. 37314918 10.1016/j.xpro.2023.102351 PMC10277608

[11] W. Minor , M. Cymborowski , Z. Otwinowski , and M. Chruszcz , “HKL‐3000: The Integration of Data Reduction and Structure Solution–From Diffraction Images to an Initial Model in Minutes,” Acta Crystallographica Section D, Biological Crystallography 62, no. Pt 8 (2006): 859–866. 16855301 10.1107/S0907444906019949

[12] P. D. Adams , P. V. Afonine , G. Bunkoczi , et al., “PHENIX: A Comprehensive Python‐Based System for Macromolecular Structure Solution,” Acta Crystallographica Section D, Biological Crystallography 66, no. Pt 2 (2010): 213–221. 20124702 10.1107/S0907444909052925 PMC2815670

[13] P. Emsley and K. Cowtan , “Coot: Model‐Building Tools for Molecular Graphics,” Acta Crystallographica. Section D, Biological Crystallography 60, no. Pt 12 Pt 1 (2004): 2126–2132. 15572765 10.1107/S0907444904019158

[14] M. van Kempen , S. S. Kim , C. Tumescheit , et al., “Fast and Accurate Protein Structure Search With Foldseek,” Nature Biotechnology 42, no. 2 (2023): 243–246. 10.1038/s41587-023-01773-0 PMC10869269 37156916

[15] L. Holm , A. Laiho , P. Toronen , and M. Salgado , “DALI Shines a Light on Remote Homologs: One Hundred Discoveries,” Protein Science 32, no. 1 (2023): e4519. 36419248 10.1002/pro.4519 PMC9793968

[16] N. A. Baker , D. Sept , S. Joseph , M. J. Holst , and J. A. McCammon , “Electrostatics of Nanosystems: Application to Microtubules and the Ribosome,” Proceedings of the National Academy of Sciences of the United States of America 98, no. 18 (2001): 10037–10041. 11517324 10.1073/pnas.181342398 PMC56910

[17] B. Yariv , E. Yariv , A. Kessel , et al., “Using Evolutionary Data to Make Sense of Macromolecules With a "Face‐Lifted" ConSurf,” Protein Science 32, no. 3 (2023): e4582. 36718848 10.1002/pro.4582 PMC9942591

[18] D. Perez‐Mendoza , D. Bertinetti , R. Lorenz , M. T. Gallegos , F. W. Herberg , and J. Sanjuan , “A Novel C‐Di‐GMP Binding Domain in Glycosyltransferase BgsA Is Responsible for the Synthesis of a Mixed‐Linkage Beta‐Glucan,” Scientific Reports 7, no. 1 (2017): 8997. 28827694 10.1038/s41598-017-09290-2 PMC5567048

[19] L. Xu , L. Xin , Y. Zeng , et al., “A Cyclic Di‐GMP‐Binding Adaptor Protein Interacts With a Chemotaxis Methyltransferase to Control Flagellar Motor Switching,” Science Signaling 9, no. 450 (2016): ra102. 27811183 10.1126/scisignal.aaf7584

